# Improving ncRNA family prediction using multi-modal contrastive learning of sequence and structure

**DOI:** 10.1093/bioinformatics/btae640

**Published:** 2024-10-26

**Authors:** Ruiting Xu, Dan Li, Wen Yang, Guohua Wang, Yang Li

**Affiliations:** College of Computer and Control Engineering, Northeast Forestry University, Harbin 150040, China; College of Computer and Control Engineering, Northeast Forestry University, Harbin 150040, China; International Medical Center, Shenzhen University General Hospital, SZU 518055, China; College of Computer and Control Engineering, Northeast Forestry University, Harbin 150040, China; College of Computer and Control Engineering, Northeast Forestry University, Harbin 150040, China

## Abstract

**Motivation:**

Recent advancements in high-throughput sequencing technology have significantly increased the focus on non-coding RNA (ncRNA) research within the life sciences. Despite this, the functions of many ncRNAs remain poorly understood. Research suggests that ncRNAs within the same family typically share similar functions, underlining the importance of understanding their roles. There are two primary methods for predicting ncRNA families: biological and computational. Traditional biological methods are not suitable for large-scale data prediction due to the significant human and resource requirements. Concurrently, most existing computational methods either rely solely on ncRNA sequence data or are exclusively based on the secondary structure of ncRNA molecules. These methods fail to fully utilize the rich multimodal information available from ncRNAs, thereby preventing them from learning more comprehensive and in-depth feature representations.

**Results:**

To tackle these problems, we proposed MM-ncRNAFP, a multi-modal contrastive learning framework for ncRNA family prediction. We first used a pre-trained language model to encode the primary sequences of a large mammalian ncRNA dataset. Then, we adopted a contrastive learning framework with an attention mechanism to fuse the secondary structure information obtained by graph neural networks. The MM-ncRNAFP method can effectively fuse multi-modal information. Experimental comparisons with several competitive baselines demonstrated that MM-ncRNAFP can achieve more comprehensive representations of ncRNA features by integrating both sequence and structural information. This integration significantly enhances the performance of ncRNA family prediction. Ablation experiments and qualitative analyses were performed to verify the effectiveness of each component in our model. Moreover, since our model is pre-trained on a large amount of ncRNA data, it has the potential to bring significant improvements to other ncRNA-related tasks.

**Availability and implementation:**

MM-ncRNAFP and the datasets are available at https://github.com/xuruiting2/MM-ncRNAFP.

## 1 Introduction

Non-coding RNAs (ncRNAs) are a category of RNA molecules that do not encode proteins. However, mounting evidence suggests that a substantial proportion of ncRNAs are functional and perform various regulatory roles within cells (Fu 2014). After several decades of research, ncRNAs have been acknowledged as indispensable functional components in biology, playing critical roles in a variety of biological processes and gene expression regulation (Srijyothi *et al.* 2018). Despite this, their precise role remains elusive. They appear to encompass a concealed layer of internal signaling that governs various levels of gene expression during physiological(Zhang *et al.* 2024) and developmental processes. These include chromatin structure, epigenetic memory, transcription, RNA splicing, editing, translation, and turnover.

Regulatory RNA networks control many complex traits, are involved in diseases, and represent an uncharted realm of genetic variation within and across species (Mattick and Makunin 2006, Wang *et al.* 2013). Due to their importance, ncRNAs are increasingly being recognized in the biomedical field (Bartoszewski and Sikorski 2018). They can be divided into long ncRNAs and small ncRNAs based on their nucleotide length. Functionally, ncRNAs can be classified as housekeeping ncRNAs [e.g. ribosomal RNA (rRNA) and transfer RNA (tRNA)] or regulatory RNAs. Long ncRNAs regulate cell cycle and differentiation (Wang and Guo 2019, Bridges *et al.* 2021), while circular RNAs also have regulatory functions (Ponting *et al.* 2009). NcRNAs make up approximately 78% of all RNA and primarily function in ribosome assembly and protein synthesis. According to the Human Genome Project, only about 3% of the genome sequence encodes proteins, while the majority produces ncRNAs (Costa 2010). Given their abundance, accurately identifying large numbers of ncRNAs remains a significant challenge in biomedical research (Fiannaca *et al.* 2017).

Methods for predicting ncRNA families predominantly fall into two categories: biological and computational (Creux *et al.* 2023). While biological prediction methods necessitate substantial manpower and resources, rendering them unsuitable for large-scale ncRNA family prediction, computational methods are indispensable for the efficient large-scale prediction of ncRNA families. The current computational methods can be bifurcated into two types. The first type exclusively relies on the sequence information of ncRNA molecules for feature learning and classification. For instance, the methods proposed by Noviello *et al.* (2020) and NCYPred (Lima *et al.* 2023) solely utilize primary RNA sequence information to predict short ncRNA families. However, this approach may necessitate longer sequences, thereby resulting in decreased accuracy and precision. ncRDeep (Chantsalnyam *et al.* 2020), on the other hand, employs convolutional neural networks as the primary prediction model. However, the simplicity of the model structure may constrain its ability to capture deeper representations, thereby affecting its accuracy in handling large datasets. A notable multi-feature fusion approach is MFPred (Chen *et al.* 2023a). MFPred comprises two modules: the first module employs four encoding methods for ncRNA sequences, which subsequently extract the sequence features in the ncRNA sequence, and the second concatenates the features extracted by the four methods for multi-feature fusion.

Studies have shown that the secondary topological structure of ncRNA molecules is important for their functions (Fabbri *et al.* 2019). However, most computational methods only consider sequence information and ignore structural information, resulting in incomplete feature learning. The second type of computational methods recognizes the importance of secondary structures in predicting ncRNA families: graph kernels are used to represent ncRNA secondary structures, and sparse vectors are generated for classification by support vector machines [e.g. EDeN (Navarin and Costa 2017), RNAGCN (Deng *et al.* 2022)]. Although these methods focus on the structural information of ncRNAs, they inevitably lose sequence information.

Other researchers suggest to integrate both sequence and structural information, e.g. the “reading frames” model (Wu *et al.* 2016), which represents the sequence information as a triplet of the central nucleotide and its flanking secondary structures in dot-bracket notation (Antczak *et al.* 2018). While this approach emphasizes the role of the secondary structure, the direct concatenation of input features may lead to correlation problems and hinder deep learning. Nevertheless, these approaches underline the complementary nature of secondary structures for family prediction tasks.

With the rapid development of pre-trained models in NLP (Han *et al.* 2021), it is promising to treat genetic sequences as text and mine rich latent information from them. For example, DNABERT (Ji *et al.* 2021) has achieved great success in DNA research. However, there is still a lack of effective methods for integrating ncRNA sequences and structural information with pre-trained models in ncRNA research. Motivated by these observations, we proposed MM-ncRNAFP, a multi-modal contrastive learning framework for ncRNA representation learning and ncRNA family prediction.

The MM-ncRNAFP framework consists of two parallel components starting from sequence modeling. Similar to DNABERT, we fine-tuned a pre-trained BERT model on a large mammalian ncRNA dataset. We used the k-mer sequence-encoding method that concatenates adjacent nucleotides into subsequences of length k. MM-ncRNAFP can capture the type, quantity, and order of nucleotides in ncRNA sequences (Chor *et al.* 2009, Liu *et al.* 2013). During pre-training, we employed self-supervised learning to investigate the syntax and semantics of the ncRNAs. To model the structure of ncRNAs, we utilized graph neural networks to represent each ncRNA molecule as a graph, thereby obtaining a graph-level representation. Contrastive learning was subsequently applied to integrate the structural features derived from the graph neural networks with the sequence features extracted by BERT. Following this, an attention mechanism was employed to amalgamate the features from both models. Finally, we fine-tuned the pre-trained weights for the family classification tasks. We used the family prediction loss and contrastive learning loss as the final objective functions, updating the parameters of both models simultaneously. We evaluated our framework using an ncRNA dataset from the RNACentral Database. The results indicate that the proposed method surpasses previous methods in terms of precision, recall, F1-score, MCC, and ACC. Notably, MM-ncRNAFP can be extended to tasks beyond ncRNA family prediction, demonstrating its potential for other ncRNA-related activities such as structure prediction (Reuter and Mathews 2010), functional annotation, and interaction network analysis.

## 2 Materials and methods

**Figure 1.**
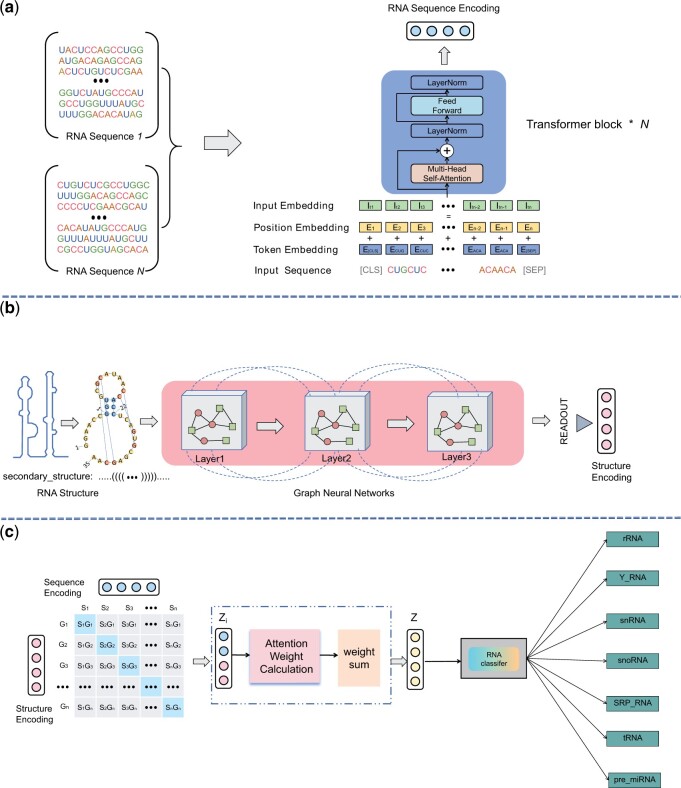
Overall framework for ncRNA family prediction (MM-ncRNAFP). (a) Non-coding RNA sequence representation learning. (b) Non-coding RNA structure representation learning. (c) Contrastive learning and family prediction.

The overall architecture of the proposed model for ncRNA family prediction is illustrated in Fig. 1. The model is composed of three modules: ncRNA sequence representation learning, ncRNA structure representation learning and ncRNA family prediction. Firstly, we pretrained BERT on a large-scale mammalian ncRNA dataset containing 190 000 first-order sequence structures. Then, we used a graph neural network to model the secondary structure of ncRNA and combined it with the BERT model by contrastive learning, which enabled the interaction between features extracted by two models. Finally, an attention mechanism was used to fuse these two types of features and then input into the final task of ncRNA family prediction.

### 2.1 Non-coding RNA sequence representation learning

In this formulation, we treat the ncRNA sequences as text and use BERT to encode in the pre-training stage with the masked language model. The ncRNA sequences are preprocessed using the k-mer method, where each k-mer (subsequence of length k) is represented as a word. The vocabulary is constructed based on all possible k-mers. A specific proportion of tokens are randomly masked and replaced with a unique [mask] token, thereby simulating the process of masking and predicting tokens during pre-training. The multi-head attention mechanism is employed within the model to extract contextual information as follows:

(1) $$\text{MultiHead}\left(Q,K,V\right)=\mathrm{concat}\left({\text{head}}_{1},\ldots ,{\text{head}}_{h}\right)W^{O}$$

(2) $${\text{head}}_{i}=\text{Attention}\left(QW_{i}^{Q},KW_{i}^{K},VW_{i}^{V}\right)$$

(3) $$\text{Attention}\left(Q,K,V\right)=\mathrm{softmax}\left(\frac{QK^{}T}{\sqrt{d_{k}}}\right)V$$

where $W_{i}^{Q}$, $W_{i}^{K}$, $W_{i}^{V}$, and $W^{O}$ are parameter matrices. Q, K, and V stand for Query, Key, and Value. The self-attention mechanism enabled BERT to encapsulate the core semantic and syntactic features of non-coding RNA sequences during its pre-training phase. We employ 190 000 primary sequences of ncRNA from RNACentral as the dataset for model pre-training. We then fine tune the model on family prediction task as shown in Fig. 1a.

### 2.2 Non-coding RNA structure representation learning

Besides the sequence structure, we also incorporate the modeling of ncRNAs’ secondary structure. The secondary structure of non-coding RNA (ncRNA) sequences offers valuable insights into their topological configuration and base-pair interactions. The secondary structure of non-coding RNA (ncRNA) can be represented in graphical form, wherein vertices signify individual nucleotides and edges denote their interactions. Graph Convolutional Networks (GCNs) have gained prominence as a potent instrument for deciphering the topological attributes and base pairing of ncRNA sequences (Xu *et al.* 2018). GCNs are adept at encapsulating the local context of nucleotides and disseminating information throughout the sequence, a process essential for comprehending the RNA’s secondary structure. GCNs, through feature embedding and neighborhood aggregation, can capture the patterns that are unique to RNA secondary structures. The multi-layered convolutional architecture of these networks facilitates the learning of both local and global contexts simultaneously, thereby improving the model’s predictive accuracy for the 3D structure of ncRNA molecules.

In this context, an ncRNA can be represented as a graph G=(V,E), where *V* signifies the set of nodes that represent the nucleotides within the ncRNA molecule, and *E* denotes the set of edges that symbolize various interactions between these nodes. Each nucleotide node *u* in an ncRNA graph is linked to a feature vector $X_{u}\in R^{d}$, which encompasses pertinent information such as the current and neighboring nucleotide categories, whether it belongs to nodes forming pseudoknots, and a series of physical or chemical characteristics. ncRNA graphs are capable of preserving fine-grained information that is essential for comprehending the structure and function of ncRNA molecules. The edges of an ncRNA sequence connect adjacent nodes within an ncRNA molecule, effectively representing the order of nucleotides and capturing the linear arrangement of the primary structure of the ncRNA molecule. This sequence information is crucial for understanding the primary structures of ncRNA molecules (Wang *et al.* 2009). Numerous studies have demonstrated that the secondary topological structures of ncRNA molecules significantly influence their functions (Svoboda and Cara 2006). Consequently, we also establish structural edges to represent the edges that form the pseudoknots. The composition of these structural edges is derived from the rules of dot bracket notation.

For each nucleotide $u_{i}$ in the graph, we initialize its representation as $h_{i}^{0}$. Then, we compute and update each node representation layer-wise using the *k*-layer GNN as follows: The updated hidden state $h_{i}^{k+1}$ for node $u_{i}$ at layer k+1 is computed as follows: where σ is the activation function, $N(u_{i})$ is the set of neighboring nodes of $u_{i}$, $c_{ij}$ is the weight of edge $e_{ij}$, and $W^{k}$ is the weight matrix at layer k. Here, $N(u_{i})$ is the set of neighboring nodes for node $u_{i}$, and $c_{ij}$ is the normalized sparsity between node $u_{i}$ and its other neighboring nodes $u_{j}$. The term $W^{k}$ is a learnable weight matrix for the k-th layer, and σ(.) is the activation function. After computing the final layer of each node representation, we obtain a graph-level representation $G_{n}$ of the n-th ncRNA by readout operation (Fig. 1b), which captures the structure information of the ncRNA.

(4) $$G_{n}=\text{Readout}(h_{i}^{k}\;|\;i\in V)$$

where Readout(·) denotes an operation that converts the representation of each node in an ncRNA graph into a comprehensive *d*-dimensional representation, thereby capturing the overall structure of the ncRNA graph.

### 2.3 Non-coding RNA family prediction

A given non-coding RNA (ncRNA) should exhibit significant similarities between its sequences and structures. Consequently, the correlations between sequences and secondary structures of identical ncRNAs are enhanced by employing a contrastive learning framework in our designed framework.

#### 2.3.1 Multi-modal contrastive learning

In particular, contrastive learning is a bridge between the two different modes (sequence and structural features) of ncRNA. It improves feature fusion by minimizing the distances of different modalities among identical ncRNAs and maximizing the distances among different ncRNAs as follows:

(5) $$L_{S}=-\frac{1}{N}\sum_{i=1}^{N}\;\text{log}\;\frac{\;\text{exp}(S^{T}G/\delta )}{\sum_{j=1}^{N}\;\text{exp}\;((S^{T}G/\delta )},$$

(6) $$L_{G}=-\frac{1}{N}\sum_{i=1}^{N}\;\text{log}\;\frac{\;\text{exp}(G^{T}S/\delta )}{\sum_{j=1}^{N}\;\text{exp}\;(G^{T}S/\delta )},$$

(7) $$L_{\text{contrastive}}=\frac{1}{2}(L_{S}+L_{G})$$

where δ is the temperature parameter, which is a hyperparameter.

Following the application of contrastive learning to sequence and structure representations, we employ an attention mechanism to amalgamate these two forms of representation. Initially, both sequence and structural features are projected into a shared latent space.

(8) $$Z_{i}=\mathrm{ReLU}(W_{s}S_{i}+W_{g}G_{i})$$

where $S_{i}$ and $G_{i}$ represent sequence features and structural features of the ncRNA, respectively, $W_{s}$ and $W_{g}$ are weight matrices.

Finally, the self-attention mechanism is utilized to ascertain the contribution of each feature dimension during the fusion process. The attention weights were calculated using the following formula:

The value of $\alpha_{i}$ is calculated as follows:

(9) $$\begin{array}{c} \alpha_{i}=\frac{e^{w_{\alpha }^{T}}z_{i}}{\sum_{j=1}^{N}e^{w_{\alpha }^{T}}z_{j}}\\ Z=\sum_{i=1}^{N}\alpha_{i}z_{i} \end{array}$$

Equation (9) represents a summation of terms, where each term is the product of an alpha coefficient and a z value. The variable N denotes the total number of terms in the summation, $Z_{i}$ denotes the final representation of ncRNA, $w_{\alpha }$ is the attention weight parameter, (Tex translation failed) is the number of feature dimensions, and $\alpha_{i}$ is the attention weight of the *i*th feature dimension.

#### 2.3.2 Family prediction

The fusion representations of sequences and structures are used for family prediction. We treat the family prediction as a multiclass classification task and train a multilayer perceptron model accordingly. To further integrate the features learned from both modalities, we design a unified loss function that combines the loss of contrastive learning with the loss of classification task, which can help the model better learn the correlation between sequence and structural features.

First, the loss of classification task is cross-entropy loss:

(10) $$L_{\text{classification}}=-\frac{1}{N}\sum_{i=1}^{N}\sum_{j=1}^{C}y_{i,j}\;\text{log}({\hat{y}}_{i,j})$$

where $y_{i,j}$ is the label corresponding to the *j*th class for the *i*th sample. ${\hat{y}}_{i,j}$ is the model-predicted probability of the *i*th sample being in the *j*th class. The loss function compares the model-predicted probability distribution to the true label distribution.

The final loss of the model is a weighted sum of two losses:

(11) $$L_{\text{total}}=\alpha \times L_{\text{classification}}+\beta \times L_{\text{contrastive}}$$

where (α+β=1), α and β are hyperparameters, adjusting the contribution of contrastive learning loss and their contribution to the entire model (Fig. 1c).

## 3 Results

In this section, we designed experiments to answer the following research questions (RQ): RQ1: Does our model achieve better performance than existing prediction methods? RQ2: Is the fused secondary structure strategy beneficial for enriching representation learning of ncRNA molecules? RQ3: Does contrastive learning help integrate these two types of features? RQ4: Is pretraining BERT on large-scale unlabeled data helpful for ncRNA family prediction?

### 3.1 Datasets

**Figure 2.**
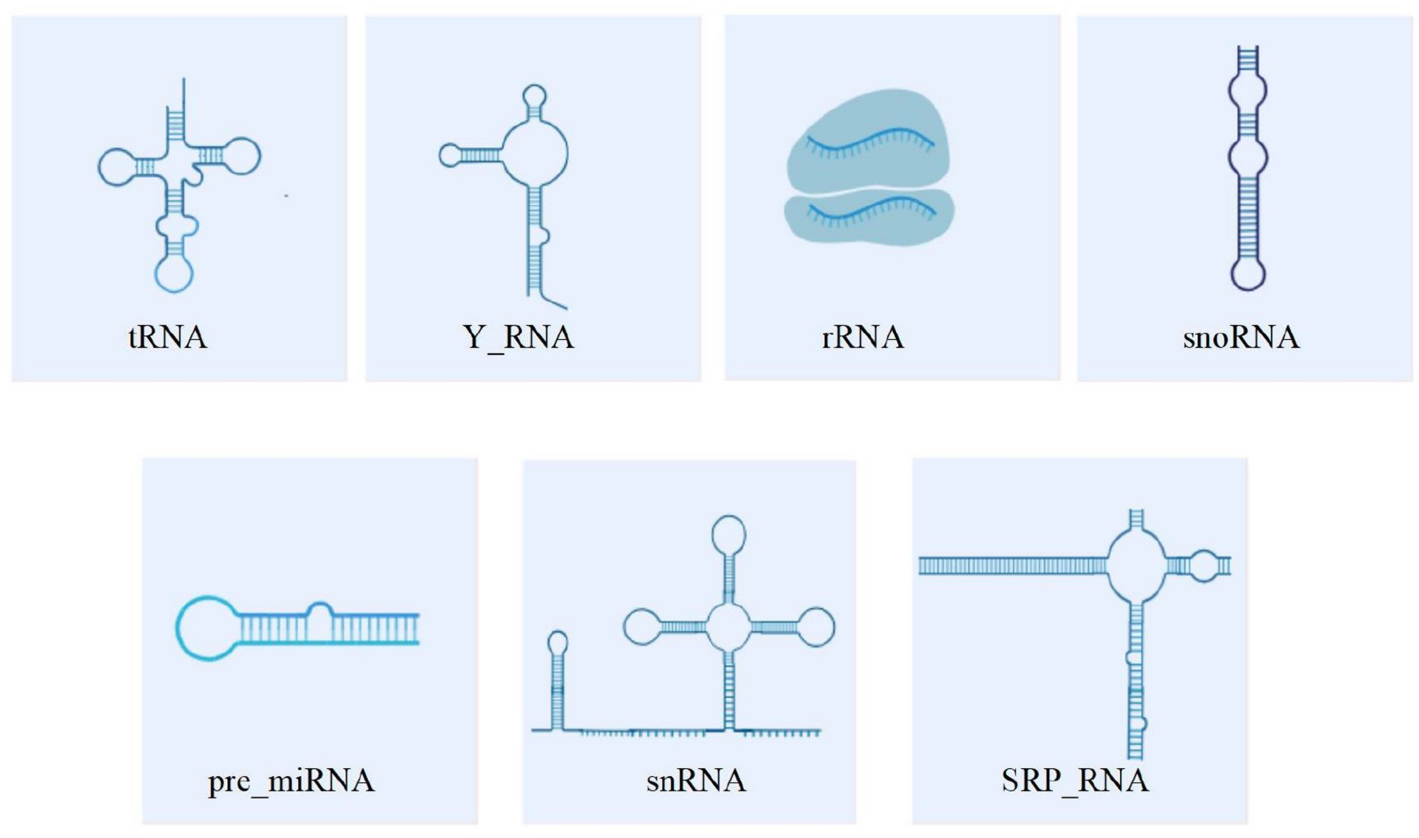
Non-coding RNA 2D structure.

This section provides an overview of our two datasets. The two datasets were constructed from several public datasets, including Rfam (Gardner *et al.* 2009), GeneCards (Safran *et al.* 2010), ENA (Leinonen *et al.* 2011), and MalaCards (Rappaport *et al.* 2017). The dot-bracket notation secondary structure representation was downloaded from the RNACentral database. The selected categories include seven important categories in ncRNA with rich secondary structure annotations in the RNACentral database (Fig. 2). Table 1 shows the detailed statistics of two datasets. The first dataset is a collection of non-coding RNA molecules from mammalian species (29 306 records), mainly house mouse. The second dataset is a collection of non-coding RNA molecules from multiple species (23 626 records), mainly non-mammalian organisms such as fungi and bacteria. Both datasets contain the same seven categories of non-coding RNA molecules.

**Table 1.** Statistics of datasets.

Type	rRNA	Y_RNA	snRNA	snoRNA	SRP_RNA	tRNA	pre_miRNA	All
**Mammalian non-coding RNA dataset**								
Training	2773	424	2405	1617	2617	3194	1228	14 258
Validation	1502	229	1108	875	1416	1729	665	7524
Test	1502	229	1108	875	1416	1729	665	7524
Total	5777	882	4621	3367	5449	6652	2558	29 306
**Multi-species non-coding RNA dataset**								
Training	1136	1178	1653	1659	2074	1979	1659	11 338
Validation	615	638	895	898	1123	1071	898	6138
Test	616	640	897	900	1124	1073	900	6150
Total	2367	2456	3445	3457	4321	4123	3457	23 626

**Rfam** is a specialized database designed for the storage, annotation, and analysis of non-coding RNA (ncRNA) sequences. These sequences encompass a variety of ncRNA families and structured RNA elements, including RNA domains and units. The RNA group at EMBL developed and maintains RFAM, which offers tools for annotating, searching, and analyzing RNA sequences and structures. This resource plays a significant role in advancing our understanding of RNA biology and functional research.

**GeneCards** is an online gene database that provides extensive genetic information to researchers and medical professionals. It consolidates diverse genomic data including gene names, aliases, locations, functions, expression patterns, associated diseases, pathways, and protein interactions. GeneCards offers a comprehensive and user-friendly platform for gene-related research and clinical application.

**ENA** (The European Nucleotide Archive), administered by the European Bioinformatics Institute, is a prominent database for nucleotide sequences. In conjunction with GenBank and DDBJ, it gathers, archives, and disseminates nucleotide sequence data, inclusive of DNA and RNA sequences, globally.

**RNACentral** is a comprehensive database designed for the storage and retrieval of ncRNA sequences globally. Acting as a centralized resource, RNACentral encompasses RNA sequences with documented secondary structure information. When such information is available, it furnishes the pertinent dot bracket notation for the current secondary structure data.

### 3.2 Experimental settings

#### 3.2.1 Baselines

We choose a group of competitive baselines for the comparison of MM-ncRNAFP:

- PlncRNA-HDeep (Meng *et al.* 2021) encodes sample sequences using k-mer nucleotides and one-hot encoding, facilitating the training of long ncRNALSTM and CNN models individually, which are combined at the decision layer. Using RNA sequences as inputs, PlncRNAHDeep captures diverse information and harnesses the advantages of both long ncRNALSTM and CNN.
- ncRFP (Wang *et al*. 2021a) is an end-to-end method for predicting ncRNA families. It relies solely on sequences for the prediction task. The model comprises BiLSTM, an attention mechanism, and fully connected networks. BiLSTM and the attention mechanism are primarily responsible for encoding various ncRNAs into standardized data formats, whereas the fully connected layer is used for decoding input to perform classification.
- ncDLRES (Wang *et al.* 2021b,c) is a method for predicting ncRNA families based on sequence feature learning, using LSTM and ResNet. Compared to homologous sequence alignment methods, it reduces data requirements and broadens the scope of application.
- ncDENSE (Chen *et al.* 2023b) uses deep learning models to predict ncRNA families by extracting features from ncRNA sequences. It employs one-hot encoding to encode the bases in a sequence, which are then input into an integrated deep-learning model comprising Bi_GRU, DenseNet, and an attention mechanism. Bi_GRU extracts features with varying weights, and attention is concentrated on information with higher weights.
- MFPred primarily identifies ncRNA families by extracting features from ncRNA sequences. The model consists of three main modules. The first module uses four sequence encodings to extract and merge features from the sequences. The second module employs Bi-GRU and a feature fusion module. The third module uses the ResNetSE module to extract the local features. The main objective of this study was to predict ncRNA families by integrating multimodal information.

#### 3.2.2 Experiment setup

We use two datasets in this study. Mammalian Non-coding RNA Dataset contains 29 306 non-coding RNAs from mammals and Multi-species Non-coding RNA Dataset contains 23 626 non-coding RNAs from mixed species. The molecules were split into training, validation, and test sets in a ratio of 2:1:1 for seven categories. During pre-training, the input was non-coding RNA sequences with maximum length of 512. About 15% of k-mers were masked during training to finish the pre-training task. The total number of steps for pre-training is 200 000. The learning rate is set to 1e-4 for the first 100 000 steps and 1e-6 for the next 100 000 steps. Training stops when loss converges after 140 000 steps. The dimension of graph features and sequence features was set to 768 during the prediction phase. The Adam optimizer was employed to train the model for 10 epochs, with a learning rate of 2e-5 and weight decay of 1e-4. Data stabilization occurred around the fifth epoch. MFPred, ncDENSE, and ncDLRES reached stable results by the 50th epoch, while plncRNA-HDeep and ncRFP stabilized by the 30th epoch. Compared with other models under the same batch size and parameters, the proposed model consumed less time. Finally, the efficacy of the model was evaluated utilizing ACC, precision, recall, MCC, and F-score as performance metrics. All experiments are conducted on 12 GB 3080 GPU hardware. Our model takes <7200 s to achieve the best results, while ncDLRES, ncDENSE, and ncRFP take at least 18 000 s, and MFPred takes at least 25 200 s to achieve the comparable results. It is important to note that our model is pre-trained on 190 000 data samples of ncRNA sequences for about three days. The pre-training process can be performed only once and then used for different tasks related to non-coding RNA.

### 3.3 Experiment results

#### 3.3.1 Non-coding RNA family prediction result

The experimental results presented in Table 2 demonstrate the superiority of our proposed method, MM-ncRNAFP, over several state-of-the-art baseline methods for predicting ncRNA functions. Across both datasets, MM-ncRNAFP consistently achieves the highest performance metrics, including accuracy (ACC), F-score, MCC, precision, and recall. For Mammalian Non-coding RNA Dataset, MM-ncRNAFP attains an impressive accuracy of 0.9881, outperforming all baselines by a significant margin. Specifically, it outperforms the closest competitor, MFPred, by approximately 1.13% in accuracy, 1.59% in F-score, and 1.36% in MCC. This indicates that our model is able to accurately classify ncRNA functions with a higher degree of precision and recall, as evidenced by its higher precision (0.9862) and recall (0.9832) scores. Similarly, in Multi-species Non-coding RNA Dataset, MM-ncRNAFP maintains its lead, achieving an accuracy of 0.9804, which is the highest among all methods. It demonstrates a notable improvement over the second-best performer, ncRFP, with an increase of 0.43% in accuracy, 0.50% in F-score, and 0.51% in MCC. This consistency in high performance shows the robustness and generalization of our method across different datasets.

**Table 2.** The comparison results of MM-ncRNAFP and baseline methods.

Model	ACC	F-score	MCC	Precision	Recall
Mammalian non-coding RNA dataset					
PlncRNA-HDeep	0.9398	0.9264	0.9273	0.9296	0.9238
ncRFP	0.9714	0.9715	0.9655	0.9715	0.9714
ncDLRES	0.9165	0.8915	0.8995	0.8950	0.8963
ncDENSE	0.9598	0.9514	0.9519	0.9524	0.9531
MFPred	0.9768	0.9687	0.9721	0.9672	0.9706
MM-ncRNAFP	**0.9881**	**0.9846**	**0.9857**	**0.9862**	**0.9832**
Multi-species non-coding RNA dataset					
PlncRNA-HDeep	0.9682	0.9693	0.9627	0.9694	0.9692
ncRFP	0.9761	0.9761	0.9719	0.9763	0.9761
ncDLRES	0.8506	0.8565	0.8270	0.8754	0.8529
ncDENSE	0.9718	0.9727	0.9671	0.9745	0.9723
MFPred	0.9685	0.9696	0.9633	0.9711	0.9674
MM-ncRNAFP	**0.9804**	**0.9811**	**0.9770**	**0.9810**	**0.9812**

Bold values show the best performance for each criterion.

#### 3.3.2 Ablation study

**Figure 3.**
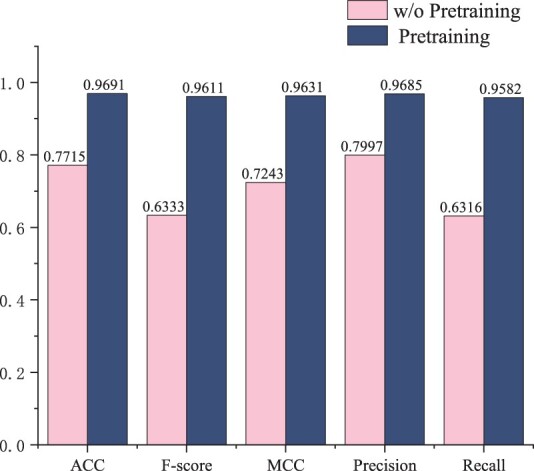
Results of the pretraining impact experiment.

This research conducted a series of ablation experiments to validate the efficacy of various components within our proposed framework. First, an experiment was set up to assess the effectiveness of the pre-trained model on ncRNA data (Fig. 3). The results indicated a significant difference between the pre-trained and non-pre-trained models, demonstrating that pre-training on a large amount of ncRNA data enhances the model’s ability to capture intrinsic features of the data and improves its predictive performance.

Besides, the effectiveness of multimodal data was also verified by the following experiments. In Table 3, seq represents the input data comprised solely of sequences, while struct_graph signifies graph data with secondary topological structures. The findings indicate that the optimal performance is achieved by integrating both structural and sequence information. Notably, sequence information alone surpassed the performance of structural information, implying that pre-training with sequence data equips the model to more effectively discern intrinsic features and patterns within the sequences.

**Table 3.** The impact of various ncRNA data construction approaches on results.

seq	struct_graph	ACC	F-score	MCC	Precision	Recall
✓	×	0.9691	0.9611	0.9631	0.9685	0.9582
×	✓	0.9524	0.9386	0.9429	0.9492	0.9337
✓	✓	**0.9881**	**0.9846**	**0.9857**	**0.9862**	**0.9832**

Bold values show the best performance for each criterion.

We conducted experiments to evaluate the effectiveness of different multimodal fusion methods, as shown in Table 4. We found that the performance when combining contrastive learning with simple concatenation was the worst, whereas using the attention mechanism alone for fusion resulted in some improvement. This suggests that if multimodal fusion methods are chosen improperly, they not only fail to enhance accuracy but may also decrease it. The best performance was achieved when contrastive learning was combined with an attention mechanism, effectively demonstrating the efficacy of contrastive learning. Moreover, compared to combining contrastive learning with concatenation, this combination mode exhibited better performance.

**Table 4.** Ablation study of the multimodal fusion module.

Model	ACC	F-score	MCC	Precision	Recall
contra+concat	0.8756	0.7689	0.8522	0.9051	0.7580
only attention	0.9686	0.9617	0.9621	0.9626	0.9609
**contra+attention**	**0.9881**	**0.9846**	**0.9857**	**0.9862**	**0.9832**

Bold values show the best performance for each criterion.

#### 3.3.3 Parameter sensitivity analysis

**Figure 4.**
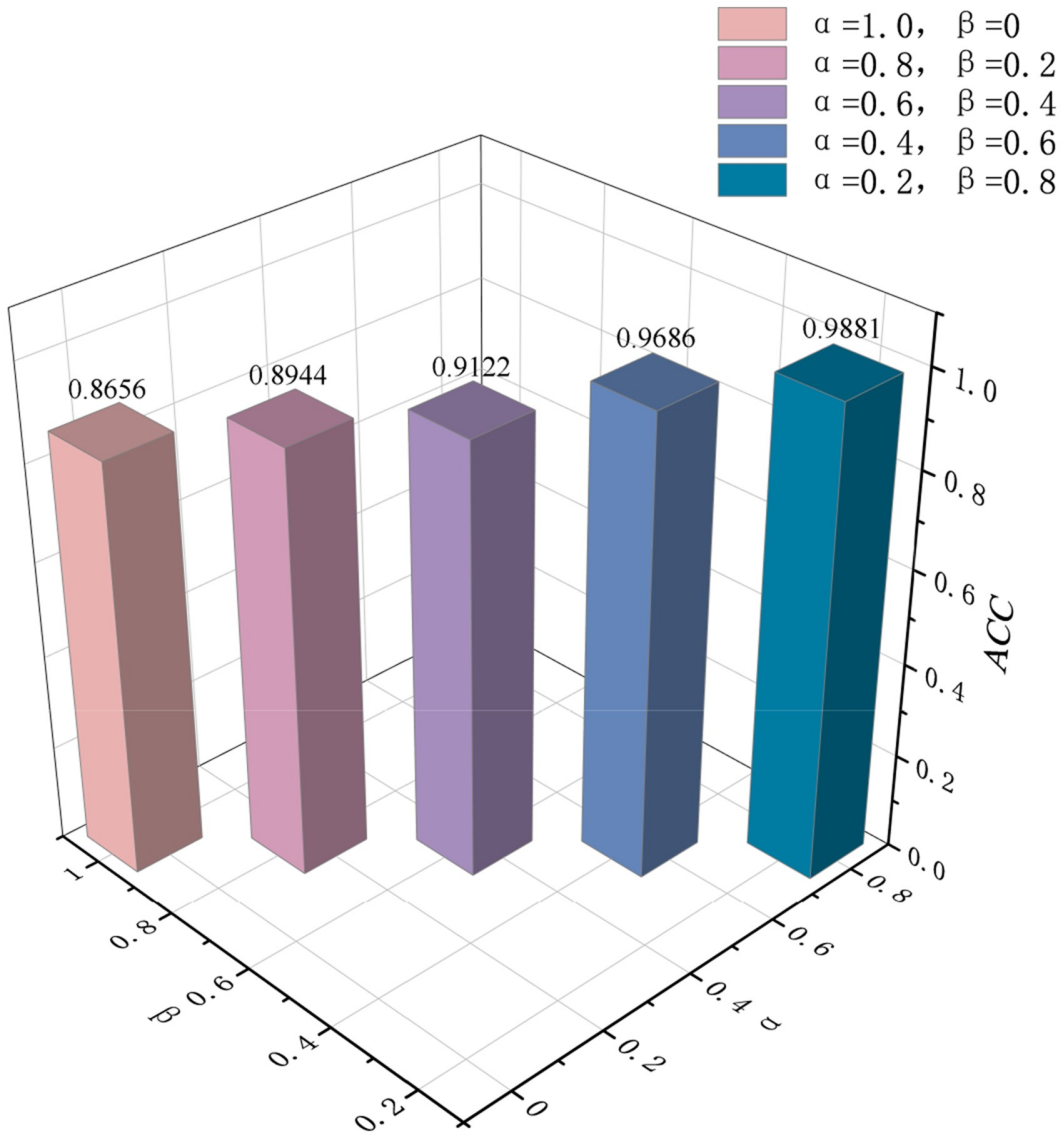
The results for varying hyperparameters α and β.

We conducted a sensitivity analysis of our model to assess its robustness and analyze the impact of the hyperparameters. Among all metrics, we chose **ACC** as the evaluation metric because it considers the performance of the model across all categories, directly reflecting the proportion of correctly classified samples. In the experiment, we analyzed the sensitivity of hyperparameters β for contrastive learning loss and α for cross-entropy loss. In this study, a grid search approach was employed to optimize hyperparameters for contrastive learning and classification tasks. All possible parameter combinations within the specified parameter space were exhaustively evaluated, and the combination yielding the best performance was selected. The results, as shown in Fig. 4, indicate that the hyperparameter setting of α+β=1. We simultaneously decreased the value of α while increasing the value of β to find the most suitable combination of hyperparameters. Figure 4, shows that setting α to 0.8 yielded the best resulted, while other values result in a decrease in the experimental results. In multi-classification tasks, the loss of the classification task is still more important than the loss of contrastive learning. However, according to the results, further reduction in the weight of the hyperparameter for contrastive learning led to a decrease in performance. This suggests that contrastive learning plays an important role in the overall task, enabling the two types of information to become closer in the feature space.

#### 3.3.4 Qualitative analysis

**Figure 5.**
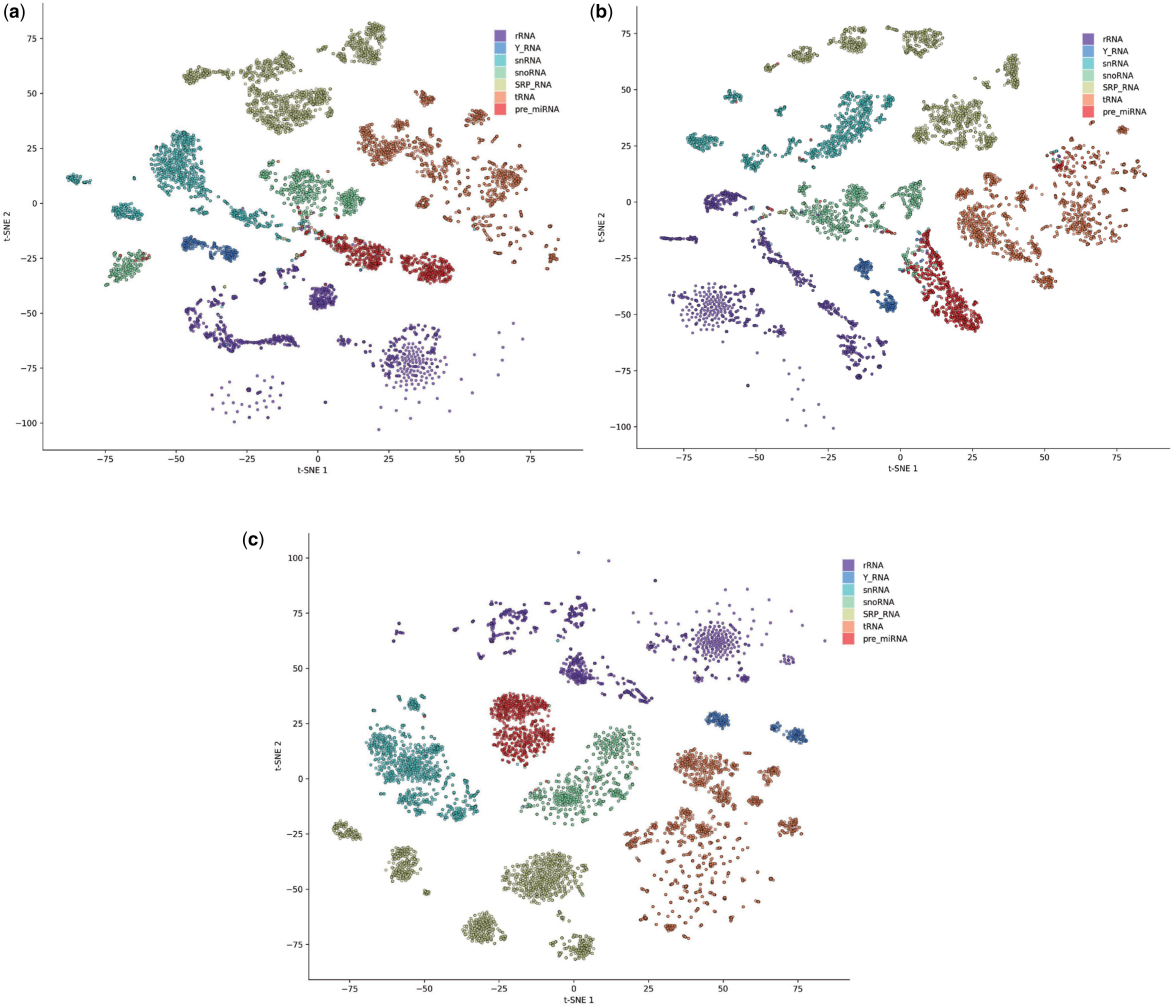
Visualization of ncRNA representation. (a) Structure feature. (b) Sequence feature. (c) Fusion feature.

To better observe the representations learned by our model for ncRNA molecules, we applied t-distributed stochastic neighbor embedding (Van der Maaten and Hinton 2008), which can visualize high-dimensional data in two or three dimensions. We selected one class from the dataset for visualization. First, we transformed high-dimensional representations of ncRNA molecules into 2D representations. We then used different colors to label the different types of ncRNA representations. The distribution plot is shown in Fig. 5. Figure 5a represents the structural features in isolation, whereas Fig. 5b exclusively illustrates the sequence features. Additionally, Fig. 5c integrates and presents the fused features derived from both sets of features. The findings suggest that the integration of these two feature sets yields the most pronounced separation for each class, demonstrating distinct clustering structures. This well-separated clustering structure suggests that our model successfully learned the feature differences among different categories and projected them into the representation space.

**Figure 6.**
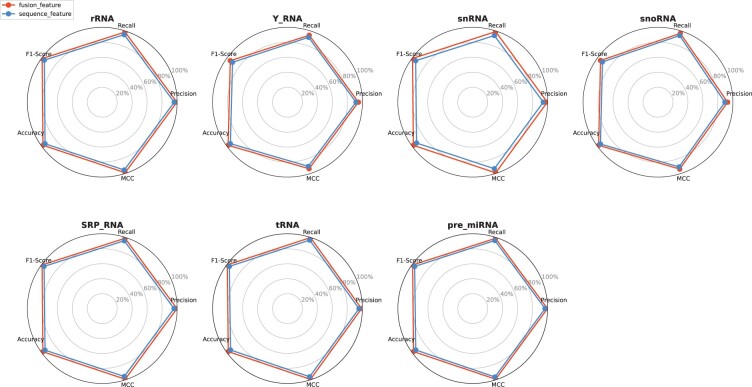
Comprehensive analysis of performance metrics for multi-class classification.

In Fig. 6, we plot the indicators of each subclass to observe which types of ncRNA are more prone to misjudgment. The red line denotes the classification performance when structural features are incorporated, while the blue line signifies the classification based solely on sequence features. Clearly, in all categories, the classification performance enhances with the inclusion of structural features, with the most substantial improvement observed in the snRNA category. We hypothesize that snRNA possesses a complex and rich secondary structure, rendering the integration of structural information particularly advantageous for this category.

## 4 Conclusion

In this study, we proposed a novel model named MM-ncRNAFP to predict ncRNA families. Through comprehensive experiments, we demonstrated that the MM-ncRNAFP achieved state-of-the-art performance in family prediction tasks. Our model innovatively regards the sequences of ncRNAs as text, and the structures of ncRNAs as graphs. Multi-modal contrastive learning effectively captures the sequence and secondary structural features of ncRNA molecules and achieved remarkable performance improvements in family prediction tasks. Additionally, because our model is pretrained on a large dataset of ncRNAs, it learns a vast array of latent representations, making it applicable not only to family prediction but also to other related ncRNA tasks.

Non-coding RNA not only plays a key role in gene regulation, but also plays a key role in the occurrence and development of various diseases(Li *et al.* 2023). Our model holds potential for future applications in disease prevention and detection. For example, recent studies have found that small nucleolar RNA(snoRNAs) plays an important role in breast cancer (Huo *et al.* 2024). Among them, SNORA7B is a kind of snoRNA, which can effectively reduce tumor occurrence by reducing the proliferation, invasion, and migration of breast cancer cells. Therefore, it is very important to accurately identify the types of non-coding RNA to promote disease research. In the future, our model can be extended to predict the association between non-coding RNA and disease.

## Data Availability

MM-ncRNAFP and the datasets are available at https://github.com/xuruiting2/MM-ncRNAFP.
